# Optimized hip-knee-ankle exoskeleton assistance reduces the metabolic cost of walking with worn loads

**DOI:** 10.1186/s12984-021-00955-8

**Published:** 2021-11-07

**Authors:** Gwendolyn M. Bryan, Patrick W. Franks, Seungmoon Song, Ricardo Reyes, Meghan P. O’Donovan, Karen N. Gregorczyk, Steven H. Collins

**Affiliations:** 1 Department of Mechanical Engineering, Stanford University, Stanford, USA; 2 Development and Engineering Center, U.S. Army Natick Soldier Research, Natick, USA

**Keywords:** Exoskeleton, Augmentation, Load-carriage, Human-in-the-loop optimization

## Abstract

**Background:**

Load carriage is common in a wide range of professions, but prolonged load carriage is associated with increased fatigue and overuse injuries. Exoskeletons could improve the quality of life of these professionals by reducing metabolic cost to combat fatigue and reducing muscle activity to prevent injuries. Current exoskeletons have reduced the metabolic cost of loaded walking by up to 22% relative to walking in the device with no assistance when assisting one or two joints. Greater metabolic reductions may be possible with optimized assistance of the entire leg.

**Methods:**

We used human-in the-loop optimization to optimize hip-knee-ankle exoskeleton assistance with no additional load, a light load (15% of body weight), and a heavy load (30% of body weight) for three participants. All loads were applied through a weight vest with an attached waist belt. We measured metabolic cost, exoskeleton assistance, kinematics, and muscle activity. We performed Friedman’s tests to analyze trends across worn loads and paired t-tests to determine whether changes from the unassisted conditions to the assisted conditions were significant.

**Results:**

Exoskeleton assistance reduced the metabolic cost of walking relative to walking in the device without assistance for all tested conditions. Exoskeleton assistance reduced the metabolic cost of walking by 48% with no load (p = 0.05), 41% with the light load (p = 0.01), and 43% with the heavy load (p = 0.04). The smaller metabolic reduction with the light load may be due to insufficient participant training or lack of optimizer convergence. The total applied positive power was similar for all tested conditions, and the positive knee power decreased slightly as load increased. Optimized torque timing parameters were consistent across participants and load conditions while optimized magnitude parameters varied.

**Conclusions:**

Whole-leg exoskeleton assistance can reduce the metabolic cost of walking while carrying a range of loads. The consistent optimized timing parameters across participants and conditions suggest that metabolic cost reductions are sensitive to torque timing. The variable torque magnitude parameters could imply that torque magnitude should be customized to the individual, or that there is a range of useful torque magnitudes. Future work should test whether applying the load to the exoskeleton rather than the person’s torso results in larger benefits.

**Supplementary Information:**

The online version contains supplementary material available at 10.1186/s12984-021-00955-8.

## Introduction

Exoskeletons could reduce the effort associated with loaded walking. Load carriage is a common task in many professions, for example military personnel will carry loads over 80% of their body weight [1]. When applied to the waist or back, each kilogram of additional load will increase metabolic cost by about 2%, and the metabolic impact increases as the load is applied further from the participant’s center of gravity [2]. In addition to increased metabolic cost, prolonged load carriage is associated with overuse injuries such as stress fractures, back strain, and knee pain [3]. Exoskeletons could assist loaded walking by reducing the user’s metabolic cost and could, over the longer term, prevent overuse injuries.

Some exoskeletons have reduced the metabolic cost of loaded walking when assisting one to two joints. Exoskeletons are typically evaluated by their ability to augment user performance, and reducing metabolic cost is an important evaluation metric [4]. Exoskeleton assistance has reduced the metabolic cost of loaded walking by up to 22% compared to walking in the device without assistance and up to 15% compared to walking without the device [5]. Many of the devices that have reduced the metabolic cost of loaded walking have assisted hip extension [6, 7], ankle plantarflexion [8–11], hip flexion and ankle plantarflexion [12, 13] or hip flexion, hip extension, and ankle plantarflexion [5, 7]. Some of these experiments applied the same mass to all participants, up to 24.5 kg in addition to the weight of the device [5–8, 11, 12, 14], while others have applied the load as a percentage of body weight, up to 30% [9, 10, 13]. Exoskeletons can reduce the metabolic cost during load carriage, but there has been limited research into optimal assistance or comparing assistance with different loads.

With increasing worn load, exoskeleton assistance has produced similar absolute metabolic reductions and decreasing metabolic reductions as a percent of control conditions. Compared to walking in the unpowered device, a hip-ankle exosuit can reduce the metabolic cost of walking by 23% (1.02 W/kg) with no load when assisting hip flexion and ankle plantarflexion [15], by 22% (1.04 W/kg) with a 7 kg load when assisting hip flexion, hip extension, and ankle plantarflexion [5] and by 15% (0.67 W/kg) with a 24 kg load when assisting hip flexion, hip extension, and ankle plantarflexion [7]. In a single-participant pilot study with bilateral ankle exoskeletons, assistance reduced the metabolic cost of walking relative to walking in the device without assistance by 33% with no load and by 15% with a 20% body weight load [9]. It is unclear if the decreasing metabolic reductions with increasing worn load is a biological trend, a result of limited actuation capabilities, or a product of assisting only one or two joints. There may be greater metabolic reductions possible when assisting the hips, knees and ankle simultaneously.

Providing assistance at all three joints (hips, knees, and ankles) may result in larger metabolic reductions for loaded walking. Biological hip extension, knee extension and ankle plantarflexion joint moments all increase with worn load [16], and optimal exoskeleton assistance may follow a similar pattern. Simulations of exoskeleton assistance when walking with a heavy load found the greatest metabolic reductions when assisting hip flexion, knee flexion, or hip abduction [17]. These simulations assume that the exoskeleton is massless, has a lossless transmission, and has unlimited torque and power capabilities. In unloaded walking, a comparison of optimized assistance for one joint, two joint, and three joint configurations found the greatest metabolic reductions when the hips, knees and ankles were assisted simultaneously [18]. The same may be true for loaded walking where optimized hip-knee-ankle exoskeleton assistance could produce greater metabolic reductions than assistance at one or two joints.

We optimized hip-knee-ankle exoskeleton assistance to reduce the metabolic cost of loaded walking. We hypothesized that exoskeleton assistance would reduce metabolic cost for all loaded conditions and that the optimized extension torques would increase with load. Three participants wore a hip-knee-ankle exoskeleton emulator [19] while we used human-in-the-loop optimization to reduce the metabolic cost of walking with no load [18, 20], while carrying 15% of body weight, and while carrying 30% of body weight. The loads were applied with a weight vest with an attached waist strap such that the majority of the load was applied to the user’s iliac crests. We measured changes in metabolic cost, muscle activity, exoskeleton assistance and kinematics across loads. We used biomechanics measurements to gain insights into the mechanisms underlying changes in metabolic rate. We expected the results of this study to be used to prescribe effective load-dependent assistance for future exoskeleton products.

## Methods

We optimized hip-knee-ankle exoskeleton assistance while participants walked at 1.25 m/s with a light load (15% of body weight) and a heavy load (30% of body weight). We optimized exoskeleton assistance for a no load condition at the same speed in previous studies [18, 20], and we re-validated the results for two participants in this study. We used human-in-the-loop optimization to minimize the metabolic cost of walking with each load.

Three able-bodied participants (1F 2M, age 26–36 years, 60–90 kg, 170–188 cm, expert users) wore a hip-knee-ankle exoskeleton emulator [19] while walking on a split-belt treadmill. The exoskeleton can apply bilateral assistance in hip flexion and extension, knee flexion and extension, and ankle plantarflexion. Participants were expert users and participated in at least one hip-knee-ankle exoskeleton optimization study before this protocol (over 70 h of hip-knee-ankle optimization experience per participant). All experiments were approved by the Stanford University Institutional Review Board and the US Army Medical Research and Materiel Command (USAMRMC) Office of Research Protections. Participants provided written and informed consent.

### Exoskeleton hardware

**Fig. 1:**
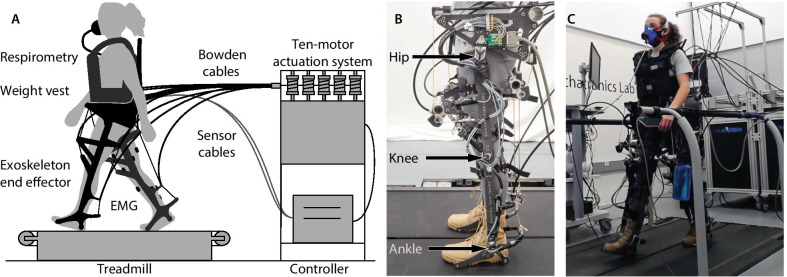
Experimental setup. **A** exoskeleton emulator system. A participant wears the hip-knee-ankle exoskeleton emulator and walks on a split-belt treadmill. Powerful, offboard motors apply joint torques through a Bowden cable transmission. **B** exoskeleton end effector. The device can assist the hips, knees, and ankles. **C** experimental setup. Metabolic rate and muscle activity are measured while a participant wears the exoskeleton end effector and walks on a split-belt treadmill

The hip-knee-ankle exoskeleton emulator can apply torque about the joints through powerful offboard motors and a Bowden cable transmission [19] (Fig. 1). The end-effector has a worn mass of 13.5 kg, and was fit to participants through length and width adjustability and interchangeable boots. This device can apply up to 200 Nm of torque in hip flexion, hip extension and ankle plantarflexion; up to 250 Nm of torque in knee extension; and 140 Nm of torque in knee flexion. It has over 4.5 kW of power available in all actuated directions.

### Exoskeleton control

**Fig. 2:**
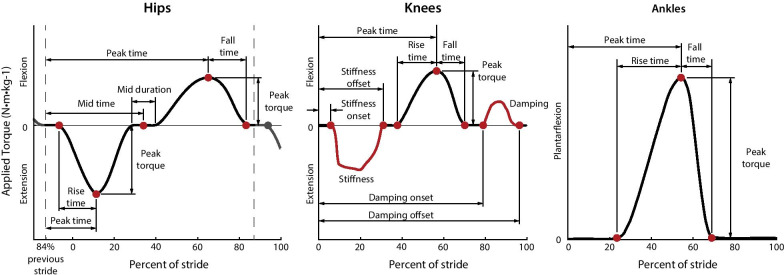
Parameterization of the hip, knee, and ankle torque profiles. Torque is defined as a function of stride time with periods of state-based torque at the knees. Hip assistance is defined by 8 parameters, knee assistance by 10 parameters, and ankle assistance by 4 parameters for a total of 22 parameters. The optimization algorithm can adjust the labeled nodes or state variables (red). The hip stride time is reset at 84% of stride to avoid discontinuities in the desired torque profile during heel strike

We applied hip, knee, and ankle exoskeleton assistance profiles as a function of stride time. These profiles were defined by 22 parameters that can be adjusted by the optimizer and consist of nodes and state variables (Fig. 2). The torque parameterization was successful in previous optimization studies with this device [18, 20]. and was inspired by biological torque data [21], effective single joint assistance [9, 22] and pilot experiments with different parameterization strategies. A more detailed derivation of the torque parameterization may be found in [18]. The profiles were generated with splines, and parameter ranges were limited for participant comfort (Additional file 1).

The hip, knee and ankle torque profiles were defined by a total of 22 parameters. The hip profile, defined by eight parameters, applied hip extension torque during early stance and hip flexion torque during swing (Fig. 2 Hips). Knee assistance applied a virtual spring during early stance, knee flexion torque near toe-off and a virtual damper during swing for a total of ten tunable parameters (Fig. 2 Knees). The two state-based periods varied on a step-to-step basis and produced step changes in desired torque at the onset and offset of the periods. The step changes were smoothed by exoskeleton actuation dynamics. Ankle assistance applied plantarflexion torque near toe-off and was defined by four parameters (Fig. 2 Ankles).

The stride time was calculated as the time between heel strikes averaged over the previous 20 strides. Ankle and knee torque profiles reset at heel strike while the hip torque profile reset at 84% of stride after heel strike. The delayed hip stride timer allowed for torque application during heel strike by preventing discontinuities in the desired torque profile.

The exoskeleton accurately applied the desired torque profiles. Torque application was controlled through closed-loop, proportional control with iterative learning [23] and velocity compensation [19]. On average, this strategy resulted in 1.7 Nm of root mean square (RMS) error (9.6% of peak torque). When no torque was commanded, the device tracked the user’s joint angles with some slack in the transmission cables so as not to apply torque. During these periods, we measured an unintended torque of 0.6 Nm on average across all joints, participants and conditions.

### Optimization protocol

We optimized exoskeleton assistance in order of increasing worn load. We found this experimental order was easiest for participants as they transitioned between load conditions. The no load condition was optimized in previous studies [18, 20]. Participants began the optimization protocol for the heavy load condition within one week of completing the light load condition validation experiment.

**Fig. 3:**
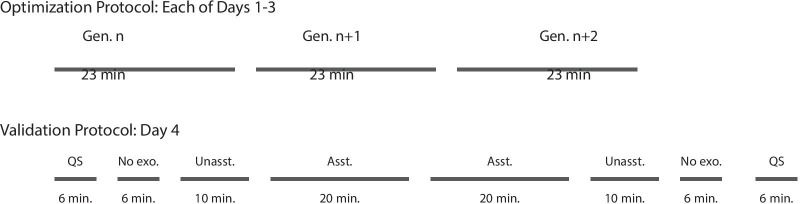
Experimental protocol. Exoskeleton assistance was optimized for three days and validated on a fourth day for each speed. Each optimization day consisted of three, 26-min-long generations with optional breaks in between each generation. On the validation day, we measured participant’s baseline metabolic rate as they stood quietly for 6 mins (QS). Participants then walked with no exoskeleton for 6 min (No exo.), in the exoskeleton with no assistance applied for 10 min (Unasst.) and then with optimized assistance for 20 min (Asst.). The protocol was then completed in the reverse order to remove effects from ordering. Participants rested at least 3 min before each of the walking conditions and at least 5 min before quiet standing conditions

For each load, we optimized exoskeleton assistance for 9 generations over the course of 3 sessions (Fig. 3). We found this was long enough for the optimization to produce consistent metabolic reductions while maintaining manageable experiment times [18, 24]. Participants fasted for 2 h before each optimization experiment to minimize the thermal effect of food within a generation, allowing for accurate comparisons over the 26 min period. Participants rested at least 1 day in between experiments. Each generation consisted of 13 conditions, and participants walked in each condition for 2 mins, resulting in 26 min of walking per generation. With 9 generations per walking speed, participants walked for at least 234 mins per optimization period. Participants could listen to podcasts while walking with an in-ear, wireless bluetooth headphone. We have found that listening to podcasts increased participant happiness and did not impact measured outcomes.

### Optimization algorithm

We used human-in-the-loop optimization to minimize the metabolic cost of walking. This strategy has been successful to reduce the metabolic cost of walking with exoskeleton assistance [9, 18, 20, 22, 24, 25]. We optimized assistance with the covariance matrix algorithm-evolutionary strategy (CMA-ES), which was successful in previous exoskeleton optimization studies [9, 18, 19, 24, 26, 27]. CMA-ES tests a generation of conditions randomly selected from a distribution defined by mean parameter values and a covariance matrix, ranks the conditions by their performance, then updates the mean and covariance matrix for the next generation based on a weighted average. We sample each condition for 2 min during which we estimate the metabolic cost through a linear dynamic model [28].

We seeded the optimization with previously optimized, participant-specific profiles to improve the likelihood of convergence. The light load condition (15% body weight) was initialized with the optimized no load parameters for each participant, and the heavy load condition (30% body weight) was initialized with the optimized parameters from the light load condition.

### Validation protocol

We validated the optimization results for each load on a separate day immediately after completing the optimization for each load and before starting the optimization for the next load (Fig. 3). Participants fasted for 4 h before validation experiments to eliminate the thermal effect of food over the whole experiment. Validation experiments required longer fasting times than the optimization experiments because metabolic rate was compared over 4 h during validation experiments and only 26 min in optimization experiments. First, the participant stood quietly for 6 min so we could measure baseline metabolic rate. Then they walked for 6 min in boots, 10 min in the device without assistance and 20 min in the device with optimized assistance. This process was then repeated in reverse to remove ordering effects (ABCDDCBA). Participants were required to rest for at least 3 min before walking conditions and at least 5 min before quiet standing so their metabolic rates would return to baseline levels. The conditions were not randomized to minimize the amount of times the participants needed to don or doff the device and to introduce conditions in ascending order of novelty. The increased time for conditions with the exoskeleton allowed the participant’s metabolic rate and kinematic adaptations to stabilize as they adjusted to walking in the device.

### Measured outcomes

We measured metabolic rate, muscle activity, ground reaction forces, exoskeleton joint angles, and exoskeleton joint torques during validation experiments. We averaged the outcomes over the last 3 min of each condition to ensure the user’s metabolic rate and gait had reached steady state. Since each condition was repeated twice, we took the average.

#### Metabolic cost

We calculated metabolic rate through indirect calorimetry. Oxygen intake and carbon dioxide output were measured with a Cosmed CPET system. Quiet standing measurements were subtracted from the walking condition measurements to calculate the metabolic cost of walking. Measurements were normalized to body mass to facilitate comparison across participants.

Participants wore a cloth or paper mask under the metabolics mask to follow COVID-19 safety protocols. We found that a cloth or paper mask under the metabolics mask slightly lowers the measured metabolic rate (Additional file 1). This did not affect comparisons across conditions.

#### Exoskeleton torques and joint angles

We estimated user kinematics by measuring the exoskeleton joint angles. Magnetic rotary encoders measured the exoskeleton hip, knee and ankle joint angles which provided reasonable approximations of user joint angles. We did not measure kinematics with motion capture because of difficulties with marker occlusion and ghost markers from reflective components on the exoskeleton.

We measured the applied torque at each exoskeleton joint. Strain gauges directly measured applied ankle torque. Load cells measured the applied force at the hips and knees, and we calculated the applied hip and knee torque by multiplying the measured force by the lever arm.

#### Exoskeleton joint power calculation

We calculated the exoskeleton joint power by multiplying the joint velocity by the applied torque. We calculated the joint velocity as the time derivative of the joint angles filtered with a zero-phase, low-pass filter with a 50 Hz cutoff frequency. Exoskeleton joint power was summed between the two legs, and the total power was the sum of the hip, knee and ankle exoskeleton joint powers.

#### Muscle activity

We measured muscle activity with surface electromyography (EMG; Delsys, Trigno). Soleus, gastrocnemius lateralis, vastus lateralis, rectus femoris, and semitendinosus activity on the participant’s right leg were measured. We were unable to measure gluteus maximus activity in the loaded conditions due to interactions of the exoskeleton and the weight vest. Measurements were bandpass filtered at 40 and 450 Hz, rectified, then low pass filtered at 10 Hz [29]. We subtracted the minimum signal value and normalized to the peak activity seen in the unassisted condition (Additional file 1). Sensor placement was similar to previous biomechanics experiments [30], with some adjustments to avoid interference with the exoskeleton structure and straps. We calculated the RMS of the average EMG profiles to evaluate muscle activity changes.

### Statistical analysis

We performed two-tailed paired t-tests and Friedman’s tests to determine significance. We performed two-tailed paired t-tests after determining that the data were normally distributed with Jarque-Bera tests and Shapiro-Wilk tests. This analysis evaluated changes from the unassisted conditions to the assisted conditions for metabolic cost magnitudes (not percent change) and RMS muscle activity. We analyzed load-related changes with Friedman’s tests for changes in positive power and metabolic rate. For all comparisons, the significance level was $\alpha =0.05$.

## Results

### Metabolic cost

**Fig. 4:**
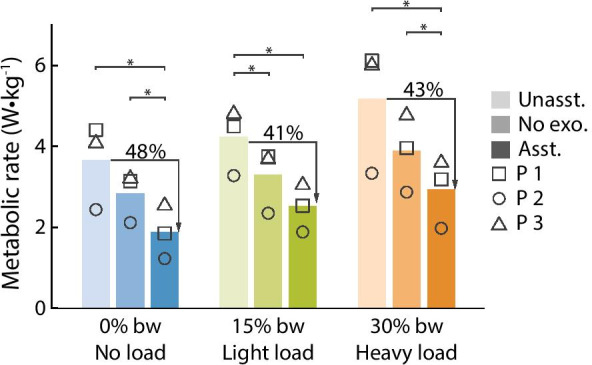
Metabolic cost of walking. The metabolic cost of unassisted walking (Unasst.), walking without the device (No exo.), and assisted walking (Asst.) with no load (blue), light load (green) and heavy load (orange). Individual metabolic scores are shown with symbols [p1 $\square$, p2 O, p3 $\triangle$]. Exoskeleton assistance significantly reduced the metabolic cost of walking relative to the unassisted condition for all three loads and relative to the no device condition for the no-load and heavy load conditions. Quiet standing has been subtracted from all costs

The metabolic cost of walking for the control conditions increased as worn load increased (Fig. 4). The metabolic cost of walking in the device without assistance increased from 3.66 W/kg in the no-load condition (range 2.44–4.40 W/kg) to 4.21 W/kg in the light load condition (range 3.28–4.85 W/kg) and to 5.17 W/kg in the heavy load condition (range 3.33–6.12 W/kg). The load related changes are significant (Friedman p = 0.0498) and similar to expected increases in metabolic rate [2, 31].

Exoskeleton assistance reduced the metabolic cost of walking for all load conditions (Fig. 4). Optimized assistance reduced the metabolic cost of walking relative to unassisted walking by 48% for the no-load condition (p1 58%, p2 50%, p3 37%, t-test p = 0.05), 41% for the light load condition (p1 44%, p2 43%, p3 36%, t-test p = 0.01) and 43% for the heavy load condition (p1 48%, p2 41%, p3 40%, t-test p = 0.04). While assistance in the no-load condition resulted in the largest percent reduction in metabolic rate, assistance in the heavy load condition resulted in the largest absolute metabolic reduction. Relative to walking in the unassisted condition, exoskeleton assistance resulted in a 1.77 W/kg reduction for the no-load condition (p1 2.55 W/kg, p2 1.21 W/kg, p3 1.54 W/kg), a 1.70 W/kg reduction for the light load condition (p1 1.96 W/kg, p2 1.39 W/kg, p3 1.76 W/kg) and a 2.24 W/kg reduction for the heavy load condition (p1 2.94 W/kg, p2 1.36 W/kg, p3 2.41 W/kg). Tables with metabolic results can be found in the Additional file 1. Because future exoskeleton products will have different masses than our testbed exoskeleton emulator, they will also have different metabolic impacts than our device, which will affect the metabolic reductions relative to walking without the device.

### Exoskeleton joint power

**Fig. 5:**
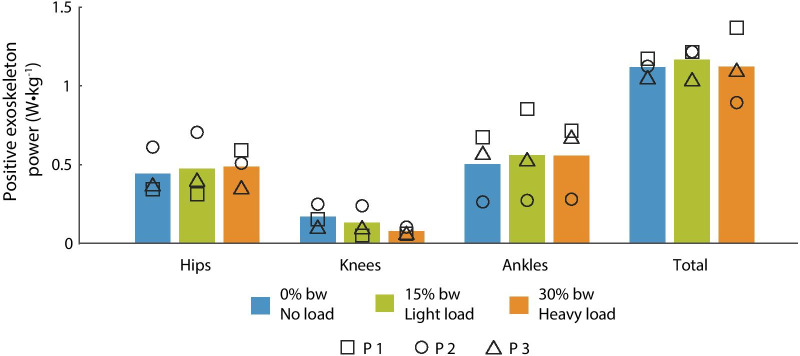
Exoskeleton positive power. Positive exoskeleton power at the hips, knees, ankles and the whole leg. The joint power was summed between the left and right legs for each joint, and the total power is the sum of the joint powers. The results for no load (blue), light load (green) and heavy load (orange) are shown, and the results for each participant are shown with symbols [p1 $\square$, p2 O, p3 $\triangle$]

Optimized exoskeleton assistance applied similar amounts of positive power for all conditions (Fig. 5). The positive power applied to the hips and ankles was similar for the three conditions. The positive power applied to the knees was substantially lower than that at the hips or ankles and decreased slightly as load increased, but this trend was not statistically significant. The exoskeleton applied a total of 1.12 W/kg with no load (range 1.06–1.17 W/kg), 1.16 W/kg with the light load (range 01.04–1.22 W/kg), and 1.12 W/kg the heavy load (range 0.89–1.37 W/kg).

### Optimized assistance

**Fig. 6:**
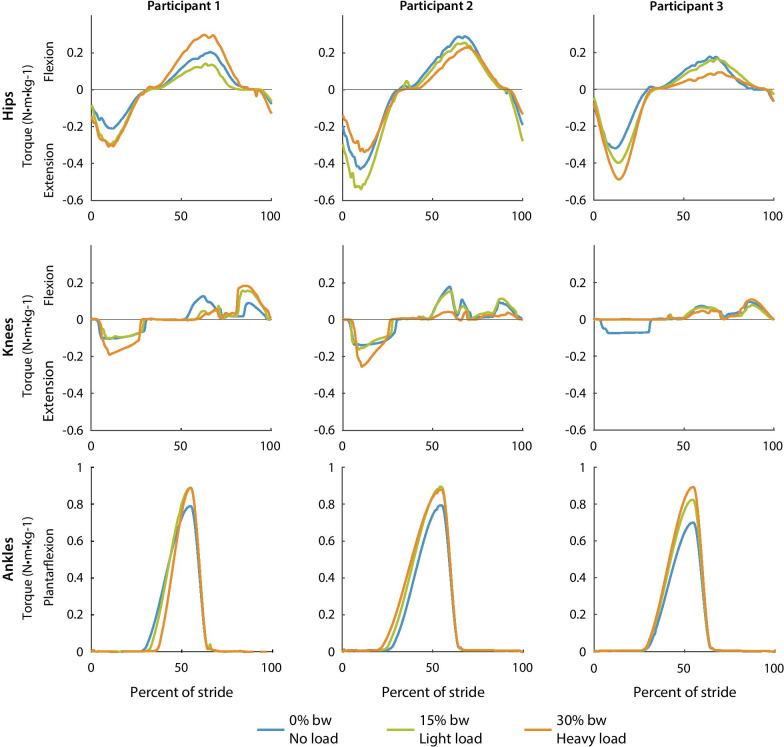
Optimized hip, knee and ankle torque profiles for each participant. The optimized torque profiles with no load (blue), light load (green), and heavy load (orange) are plotted as a percent of stride. Ankle torque magnitude was the smallest for the no-load condition and largest for the heavy load condition for all three participants. Optimized hip and knee torque magnitudes varied for each participant. Most timing parameters were similar across loads and participants. The knee torque profiles have two state-based periods which result in torque changes on a step-to-step basis

Optimized torque magnitudes varied while most optimized timing parameters were consistent across loads and participants (Table 1, Fig. 6). Optimized ankle torque magnitude increased from the no-load condition to the heavy load condition, where it optimized to the comfort limit (0.9 Nm/kg) for all three participants. The comfort limit was increased from the no-load condition (0.8 Nm/kg) to the loaded conditions (0.9 Nm/kg) because load carriage allowed for the comfortable application of larger ankle torque magnitudes (Additional file 1). Optimized torque magnitude at the hips and knees varied across participants. For example, hip extension torque increased as load increased for participant 3 but decreased in the heavy load condition for participant 2. All hip and knee parameters optimized to values within the comfort limits. The variable torque magnitudes and consistent timing parameters indicate that metabolic reductions may be sensitive to torque timing and less sensitive to torque magnitude.

**Table 1:** Minimum, average, and maximum optimized parameters over all load conditions

Hips	HE RT	HE PT	HE T	Mid. T	Mid. D	HF PT	HF T	HF FT
Min.	0.18	0.25	0.21	0.47	0.00	0.80	0.09	0.20
Avg.	0.19	0.26	0.37	0.48	0.01	0.82	0.20	0.23
Max.	0.21	0.29	0.54	0.49	0.05	0.85	0.31	0.26

Knees	k On	k	k Off	KF RT	KF PT	KF T	KF FT	b On	b	b Off
Min.	0.02	0.00	0.23	0.15	0.59	0.03	0.08	0.75	0.74	0.96
Avg.	0.03	0.01	0.28	0.17	0.60	0.10	0.10	0.78	1.42	0.98
Max.	0.04	0.03	0.30	0.20	0.61	0.20	0.11	0.81	2.19	1.00

Ankles	A T	A PT	A RT	A FT
Min.	0.71	0.55	0.20	0.17
Avg.	0.85	0.55	0.29	0.18
Max.	0.90	0.55	0.38	0.19

The timing parameters were consistent across load conditions and participants while the torque magnitudes varied. The timing parameters are reported in stride time percentage, the magnitude parameters are in Nm/kg, the stiffness parameters are reported in Nm/kg/deg., and the damping parameters are reported in Nm/kg/deg./s

Limitations in torque tracking capabilities resulted in small vibrations of applied hip torque and small, abrupt changes in knee flexion torque (Fig. 6). The errors in torque tracking are most visible during swing when joint angles are rapidly changing and the participant is not braced against the ground. The torque tracking errors were small enough that they were not noticeable to participants.

### Joint angles

**Fig. 7:**
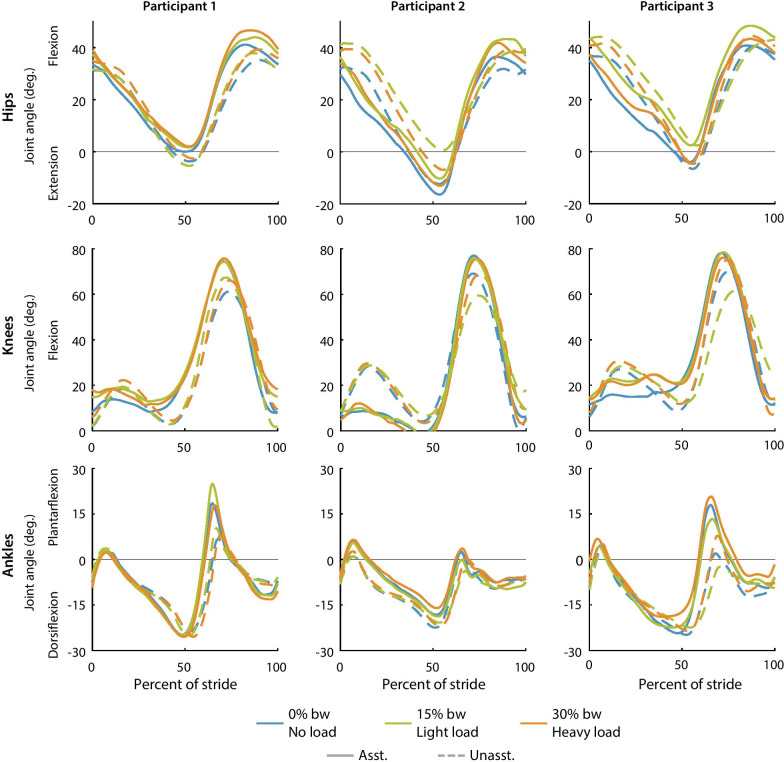
Average hip, knee and ankle joint angle trajectories for each participant. Assisted (solid) and unassisted (dashed) joint angle trajectories are shown for the no-load (blue), light load (green), and heavy load (orange) conditions. Joint angle trajectories changed slightly with load typically in the direction of exoskeleton assistance

Joint angle trajectories changed slightly with load (Fig. 7). For example, peak hip flexion during late swing was the smallest for the no-load condition and increased for the loaded conditions. This trend was consistent with and without assistance, and most other joint angle changes with load were small.

Participants walked with different gait kinematics in the assisted conditions compared to the unassisted conditions (Fig. 7). Exoskeleton assistance typically shifted the joint angle trajectories in the direction of the applied torque. For example, the knee flexion excursion during stance decreased when knee extension torque was applied and knee flexion excursion increased near toe off when knee flexion torque was applied. In some instances, participants developed unique walking patterns. For example, from 0 to 50% of stride, participant 2 walked with less knee flexion and less ankle dorsiflexion than participants 1 and 3.

### Muscle activity

**Fig. 8:**
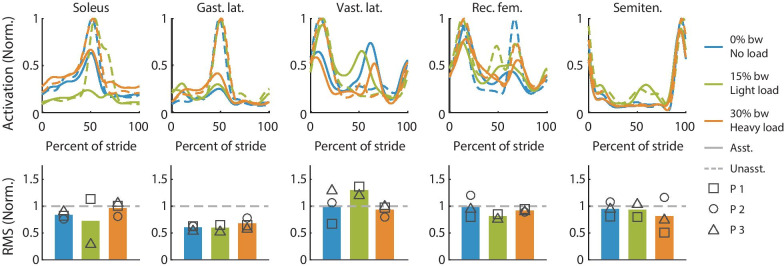
Average muscle activity profile over a stride (top row) and RMS of muscle activity (bottom row). The top row shows the averaged unassisted (dashed) muscle activity profile over a stride and the assisted (solid) no load (blue), light load (green) and heavy load (orange) conditions. The bottom row shows the RMS of the muscle activity with assistance for all load conditions. The RMS of the unassisted muscle activity is shown with the gray line (dashed). Muscle activity was normalized to the unassisted activity resulting in a peak value of 1 for unassisted walking at all loads

Exoskeleton assistance typically reduced muscle activity relative to the unassisted conditions across loads (Fig. 8). We normalized the muscle activity to the maximum value in the unassisted profile for each load condition. There was no clear trend in muscle activity changes with assistance as worn load increased. Assistance reduced the peak and RMS plantar flexor activity near toe off (soleus and gastrocnemius lateralis), knee extensor activity during stance (vastus lateralis), hip flexor activity during swing (rectus femoris), and hamstring activity during late swing (semitendinosus). Vastus lateralis activity increased around 60% of stride for all three conditions. This may be an adaptation to better use plantarflexion and hip flexion assistance, or it may be an indication of resistance to exoskeleton assistance. The light load condition muscle activity data for participant 2 was corrupted and therefore not included.

## Discussion

Exoskeleton assistance can substantially reduce the metabolic cost of walking for a wide range of carried loads. Relative to the unassisted condition, optimized exoskeleton assistance reduced the metabolic cost of walking by 48% in the no-load condition, 41% in the light load condition, and 43% in the heavy load condition. The metabolic cost reductions from this study show a marked improvement to exoskeleton assistance during load carriage. Compared to prior studies with similar load magnitudes, the reductions in metabolic cost in the light load condition are twice as large as previous successful strategies [5, 9] and those in the heavy load condition are three times larger [6–8, 11–13]. The optimized torque magnitudes found in this study are similar to the maximum abilities of leading autonomous exoskeletons (45 Nm available at the hips [32], 80 Nm at the knees [33] and 120 Nm available at the ankles [8]) and exceed the capabilities of many autonomous devices [5, 34–37]. Therefore, the improved metabolic cost reductions from this study may be a result of optimizing exoskeleton assistance for each load and participant, assisting the hips, knees and ankles simultaneously, and using exoskeleton hardware with greater torque and power capacities than state-of-the art autonomous devices.

The metabolic reduction as a percent of the control conditions slightly decreased while the absolute reduction slightly increased from the no load to the heavy load condition. Previous exoskeleton studies saw that the percent reduction decreased and the absolute reduction was similar as worn load increased [5, 7, 9, 15]. In this study, the no-load condition resulted in the largest percent reduction (48%) while the heavy load condition resulted in the largest absolute reduction (2.29 W/kg). The light load condition resulted in the smallest percent and absolute reductions (41% and 1.70 W/kg). It may be that the variable reductions in metabolic cost are due to measurement noise or random variations in participant behavior and that the mean expected values are similar for all tested conditions.

Optimized torque timing parameters were similar across participants and load conditions, while optimized torque magnitudes varied. The consistency of optimized timing parameters suggests that metabolic reductions are sensitive to the timing of torque application and that a narrow range of values are most effective. We do not believe that the initialization values contributed to the similar optimized timing parameters in this study. A wide variety of timing parameters were evaluated over the 9 generations, but alternate timing values were not selected. By contrast, optimized torque magnitude parameters did differ substantially from their initialization values. The consistent optimized timing parameters therefore appear to be a result of the optimization and not solely an effect of the initial seeds. Metabolic cost reductions may also be sensitive to ankle torque magnitude, which was largest for the heavy load condition and smallest for the no-load condition, though this is in part due to easing the maximum ankle torque limit for the loaded conditions. The torque magnitudes at the hips and knees varied across participants and load conditions. This variability suggests that exoskeleton assistance may be best when customized to the individual, or that there may be a wide variety of effective assistance strategies. Future exoskeleton experiments could present one participant with optimized assistance patterns from another participant or the average pattern across all participants in this study to differentiate between these possibilities.

Greater metabolic cost reductions may be possible by applying the worn load to the device, improved control strategies, or increased exposure to the exoskeleton. If the added load were carried directly by the exoskeleton, it may be able to more completely offload the participant, effectively applying gravity and inertia cancellation to the load [38] in addition to torques that assist body movement. Loading the device as opposed to the participant may produce greater metabolic cost reductions, but this strategy is not possible with soft exosuits or exoskeletons that assist one to two joints. Future exoskeleton products for load carriage could be designed to carry the worn load in addition to assisting the participant. Improved control strategies may allow for the comfortable application of larger torque magnitudes. In most cases in this study, ankle torque magnitude optimized to the comfort-based torque limits, which suggests that greater metabolic cost reductions may be possible with the application of larger torque magnitudes. This could be accomplished through improving exoskeleton control strategies to comfortably apply large torque magnitudes. Large torque magnitudes can easily make exoskeleton assistance difficult to walk in, so to be effective, the control strategy would need to maintain participant comfort and stability. Finally, wearing the exoskeleton more frequently for an extended period of time may lead to greater reductions in metabolic cost, as the participant may learn to take better advantage of the exoskeleton assistance.

We expect the experimental protocol from this study would be effective for naïve participants but would require longer optimization times. Naïve participants will likely experience smaller metabolic reductions initially, and after enough exposure, the metabolic reductions would increase to similar values to the results from this study. Experienced participants can comfortably walk with larger torque magnitudes than naïve participants, which may contribute to the smaller, initial metabolic reductions. In addition, participants become acclimated to walking with the structure of the device over time. We have seen that the metabolic increase from the no exoskeleton condition to the unassisted condition decreases with experience. To achieve similar metabolic results to this study, an experimental protocol with naïve participants would need to be longer to allow for sufficient exposure.

Exoskeleton assistance impacts user joint angle and muscle activity trajectories, but the long term effects are unclear. Participants varied their joint angle trajectories when walking with exoskeleton assistance, typically by shifting joint angles in the direction of the applied assistance. This could potentially lead to negative outcomes with long term use. For example, participants walked with straighter knees during stance with exoskeleton assistance which may increase the load through the knee joint. This might result in increased progression of osteoarthritis or overuse injuries. Conversely, exoskeleton assistance reduced muscle activity relative to the unassisted condition which may decrease the load through the joints resulting in a slowed progression of osteoarthritis or a decrease in overuse injuries. Future studies could evaluate the impact of long-term exoskeleton assistance use through longitudinal studies.

This study could have been improved by testing more participants, but we were limited by experiment length (over 40 h of data collection per participant) and safety concerns with the COVID-19 pandemic. A power analysis with a statistical power of 0.8, three participants and a standard deviation similar to previous exoskeleton studies (7.3% in [9]) shows that the smallest detectable change in metabolic rate is 24% [18]. The results of this study are statistically significant in part due to the large magnitude of the observed changes. The small sample size decreases the robustness to outliers, so with more participants, trends in metabolic cost with increased load could become clearer.

The optimized torque patterns identified here may inform the design of future exoskeleton products for load carriage. We found that optimized timing parameters were consistent across loads and participants while optimized magnitudes varied. This suggests that future exoskeleton products could be effective while applying the same torque timing to all participants and loads and only varying the torque magnitude. For example, naïve users may find smaller torque magnitudes effective while expert users may prefer larger torque magnitudes. The variability in the optimized torque magnitudes may indicate that there is a range of equally effective torque magnitudes. Therefore, it may be possible for future exoskeleton products to achieve similar benefits while applying the same pattern of assistance for all load carriage conditions and participants. The results from this study may provide an estimate of the torques needed for future products to deliver large metabolic cost reductions.

The results of this study may also influence future exoskeleton experiments. Future studies could investigate loading the device and applying gravity and inertia cancellation strategies. Exoskeletons could also assist hip ad/abduction, which simulations suggest could be particularly useful for load carriage [17]. Future experiments could optimize exoskeleton assistance with larger worn loads that may be more analogous to the loads carried by military personnel. We found indications that worn load might slightly interact with metabolic cost reductions, but the changes were small. Future studies could enroll more participants to increase power and target other biomechanical measurements that may be more sensitive to load carriage. For example, exoskeletons could target reducing muscle activity or joint loading to prevent injuries. In addition, the optimized torque profiles from this study may facilitate future experiments with naïve participants. Optimized torque patterns varied with load in different ways for each participant, so future studies could evaluate participant’s sensitivity to customized exoskeleton assistance.

## Conclusions

Optimized hip-knee-ankle exoskeleton assistance can substantially reduce the metabolic cost of walking with a wide range of worn loads. These metabolic reductions are notably larger than previous successful assistance strategies for load carriage, which may be due to the large device capabilities, optimized exoskeleton assistance, and simultaneously assisting the hips, knees, and ankles. The consistent optimized timing parameters suggest that metabolic reductions are sensitive to torque timing. However, the variable magnitude parameters imply that torque magnitude should be customized to the individual or that there is a range of useful torque magnitudes. Future work should test whether applying the worn load to the exoskeleton rather than the participant’s torso results in larger benefits.

## Supplementary information

**Additional file 1.** The supplementary materials include participant and validation protocol information, metabolic results, applied power, muscle activity, kinematic results, torque parameterization, parameter ranges and optimized values, torque tracking, and the impact of the cloth mask.

## Data Availability

The datasets for this study are available from the corresponding author on reasonable request.

## Abbreviations

EMG: Electromyography
RMS: Root mean square
