# Supplementary material for: Optimized hip-knee-ankle exoskeleton assistance reduces the metabolic cost of walking with worn loads
Source: J Neuroeng Rehabil. 2021 Nov 7;18:161. doi: 10.1186/s12984-021-00955-8 (PMC8572578; doi:10.1186/s12984-021-00955-8)
Supplement: Supplementary file 1 — Additional file 1. The supplementary materials include participant and validation protocol information, metabolic results, applied power, muscle activity, kinematic results, torque parameterization, parameter ranges and optimized values, torque tracking, and the impact of the cloth mask. [file 12984_2021_955_MOESM1_ESM.pdf]

1.Participant information, validation protocol 2

2. Metabolic results 3

2. Applied power 4

3. Muscle activity 5
Profiles 5
Minimum value subtraction 6

4. Kinematic results 8
Ground reaction forces 8
Stride frequency 8

5. Torque parameterization 9

6. Parameter ranges and optimized values 10
Hip parameter ranges and optimized values 10
Knee parameter ranges and optimized values 11
Ankle parameter ranges and optimized values 12

7. Torque tracking 13

8. Impact of the cloth mask 14

1.Participant information, validation protocol

For this study, we revalidated the no load condition for participants 1 and 2 to allow for a more direct comparison between conditions and participants. The original validation experiments occurred over 7 months earlier and before the implementation of Covid-19 masking protocols. The re-validation sessions accounted for any physical changes the participants underwent over the 7 month period, such as overall fitness or weight, as well as the protocol change to include the paper or cloth masks.
We did not revalidate the no load condition for participant 3 because they completed the no load validation experiment less than a month before beginning this protocol when masking protocols were already in place. All three participants underwent identical laboratory testing procedures.

Table 1. Metabolic cost in W/kg for participants 1 and 2 no load condition with the initial and revalidated results.

P1	Quiet standing	No exoskeleton	No torque	Optimized torque
Initial	1.77 ± 0.01	5.27 ± 0.06	6.17 ± 0.12	3.84 ± 0.06
Revalidation	1.63 ± 0.08	4.77 ± 0.08	6.04 ± 0.41	3.48 ± 0.10
P 2				
Initial	1.33 ± 0.02	3.88 ± 0.09	4.56 ± 0.14	2.92 ± 0.11
Revalidation	0.86 ± 0.06	2.97 ± 0.14	3.30 ± 0.01	2.09 ± 0.20

2. Metabolic results

Table 2. Metabolic cost in W/kg for the no-load condition.

	Participant information	Quiet standing	No exoskeleton	No torque	Optimized torque
P 1	60 kg, 170 cm, F	1.63 ± 0.08	4.77 ± 0.08	6.04 ± 0.41	3.48 ± 0.10
P 2	90 kg, 187 cm, M	0.86 ± 0.06	2.97 ± 0.14	3.30 ± 0.01	2.09 ± 0.20
P 3	80 kg, 182 cm, M	1.56 ± 0.02	4.82 ± 0.12	5.69 ± 0.26	4.15 ± 0.07

Table 3. Metabolic cost in W/kg for the light load condition.

	Participant information	Quiet standing	No exoskeleton	No torque	Optimized torque
P 1	60 kg, 170 cm, F	1.44 ± 0.02	5.19 ± 0.16	5.93 ± 0.15	3.97 ± 0.10
P 2	91.5 kg, 187 cm, M	1.02 ± 0.11	3.37 ± 0.21	4.30 ± 0.12	2.90 ± 0.20
P 3	80 kg, 182 cm, M	1.71 ± 0.05	5.45 ± 0.04	6.56 ± 0.02	4.81 ± 0.05

Table 4. Metabolic cost in W/kg for the heavy load condition.

	Participant information	Quiet standing	No exoskeleton	No torque	Optimized torque
P 1	60 kg, 170 cm, F	1.53 ± 0.03	5.48 ± 0.29	7.65 ± 0.01	4.71 ± 0.09
P 2	90 kg, 187 cm, M	1.14 ± 0.05	4.01 ± 0.19	4.47 ± 0.02	3.11 ± 0.12
P 3	80 kg, 182 cm, M	1.69 ± 0.13	6.51 ± 0.14	7.74 ± 0.26	5.33 ± 0.07

2. Applied power

Table 5. Positive exoskeleton power in W/kg at the hips, knees and ankles and the sum of all three.

	Hips	Knees	Ankles	Total
0%	0.44 ± 0.15	0.17 ± 0.07	0.50 ± 0.21	1.12 ± 0.06
15%	0.47 ± 0.21	0.13 ± 0.10	0.55 ± 0.32	1.16 ± 0.10
30%	0.49 ± 0.12	0.08 ± 0.02	0.56 ± 0.24	1.12 ± 0.24

Table 6. Net exoskeleton power in W/kg at the hips, knees and ankles and the sum of all three.

	Hips	Knees	Ankles	Total
0%	0.42 ± 0.14	0.05 ± 0.08	0.39 ± 0.22	0.87 ± 0.03
15%	0.45 ± 0.20	-0.01 ± 0.12	0.44 ± 0.32	0.88 ± 0.01
30%	0.47 ± 0.12	-0.07 ± 0.11	0.46 ± 0.30	0.85 ± 0.18

Table 7. Negative exoskeleton power in W/kg at the hips, knees and ankles and the sum of all three.

	Hips	Knees	Ankles	Total
0%	-0.02 ± 0.01	-0.12 ± 0.02	-0.12 ± 0.05	-0.26 ± 0.03
15%	-0.02 ± 0.01	-0.14 ± 0.05	-0.11 ± 0.07	-0.28 ± 0.10
30%	-0.02 ± 0.01	-0.15 ± 0.09	-0.10 ± 0.07	-0.27 ± 0.09

3. Muscle activity

Profiles

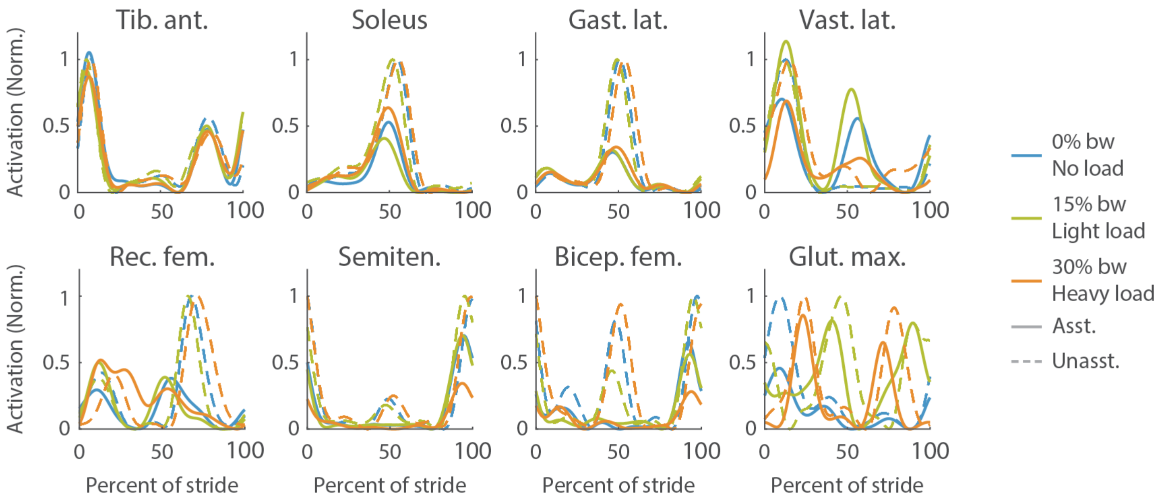

Figure 1. Muscle activity averaged over a stride for participant 1. Increased bicep femoris activity near 50% of stride is due to the sensor interacting with a thigh strap. Gluteus maximus activity with the light load and heavy load was impacted by interactions of the weight vest and exoskeleton waist strap.

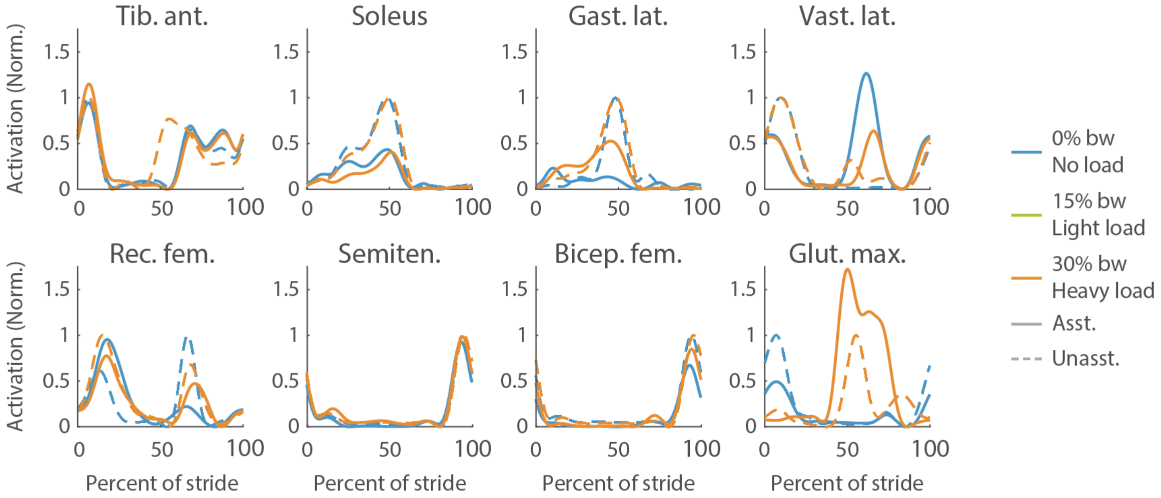

Figure 2. Muscle activity averaged over a stride for participant 2. Gluteus maximus activity with the heavy load was impacted by interactions of the weight vest and exoskeleton waist strap. The light load condition results were corrupted and not included.

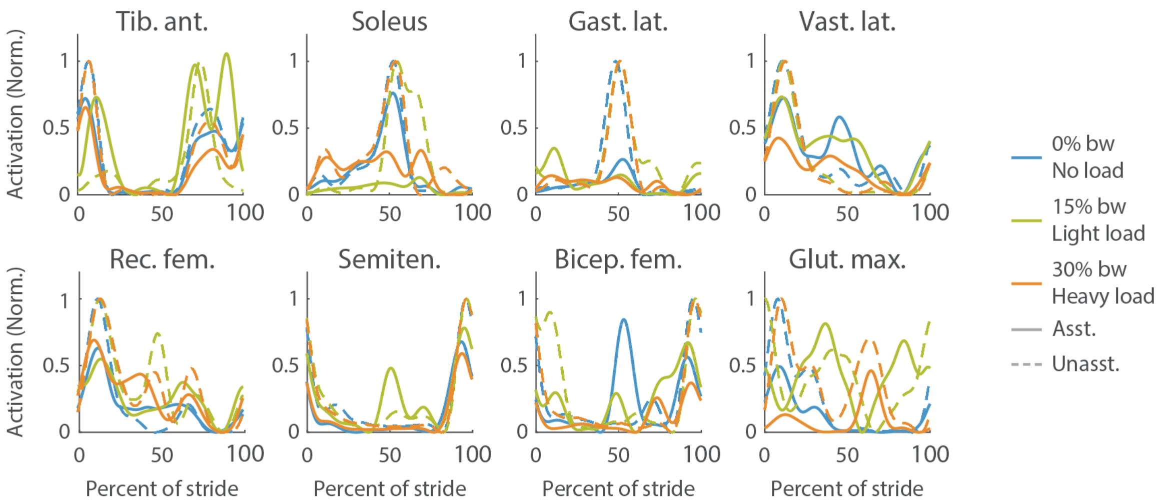

Figure 3. Muscle activity averaged over a stride for participant 3. Increased semitendinosus and bicep femoris activity near 50% of stride is due to the sensor interacting with a thigh strap. Gluteus maximus activity with the light load was impacted by interactions of the weight vest and exoskeleton waist strap. Increased gluteus maximus activity for the heavy load near 60% of stride was due to strap interactions with the sensor.

Minimum value subtraction

To process the muscle activity data, the data was bandpass filtered at 40 and 450 Hz, rectified, then low pass filtered at 10 Hz. We also subtracted the minimum signal value because high frequency noise produced a constant offset that we were unable to remove through filtering. The offset implied constant muscle activation specifically during times of rest. For example, soleus electromyography signals were non-zero during swing when it is typically quiescent. This subtraction impacted the RMS activity. In some instances, subtracting the offset produced a smaller RMS value than when the offset was not subtracted, while in other cases, the subtraction resulted in larger RMS values. Muscle activity results are plotted below with and without the minimum value subtracted.

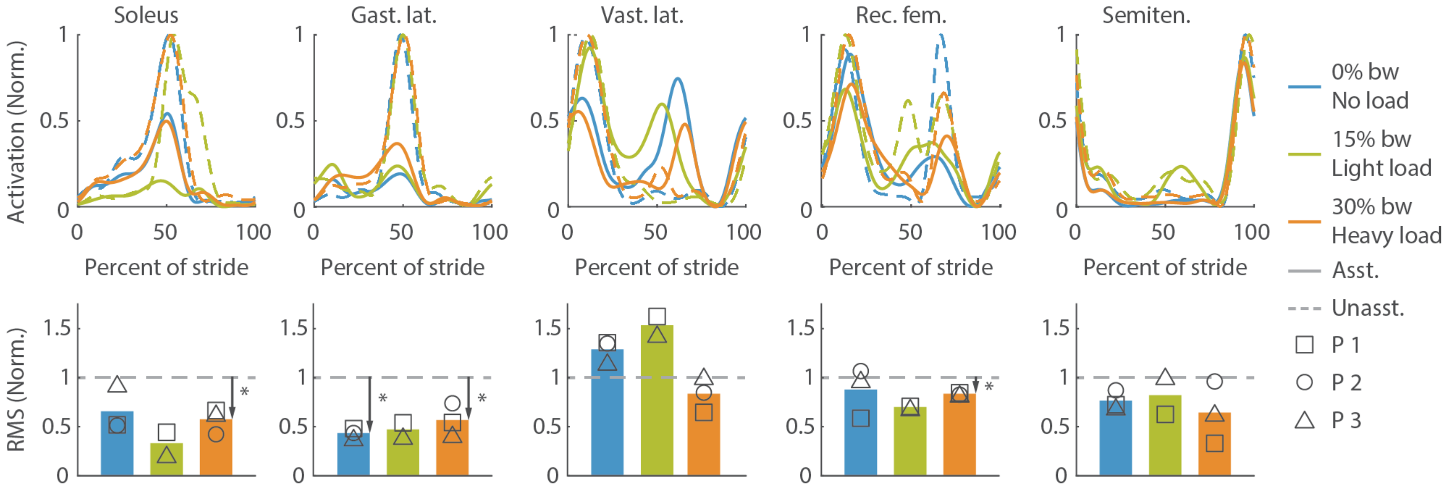

Figure 4. Average muscle activity profile over a stride (top row) and RMS of muscle activity (bottom row) with the minimum value subtracted. The top row shows the averaged unassisted (dashed) muscle activity profile over a stride and

the assisted (solid) no load (blue), light load (green) and heavy load (orange) conditions. The bottom row shows the RMS of the muscle activity with assistance for all load conditions. The RMS of the unassisted muscle activity is shown with the gray line (dashed). Muscle activity was normalized to the unassisted activity resulting in a peak value of 1 for unassisted walking at all loads.

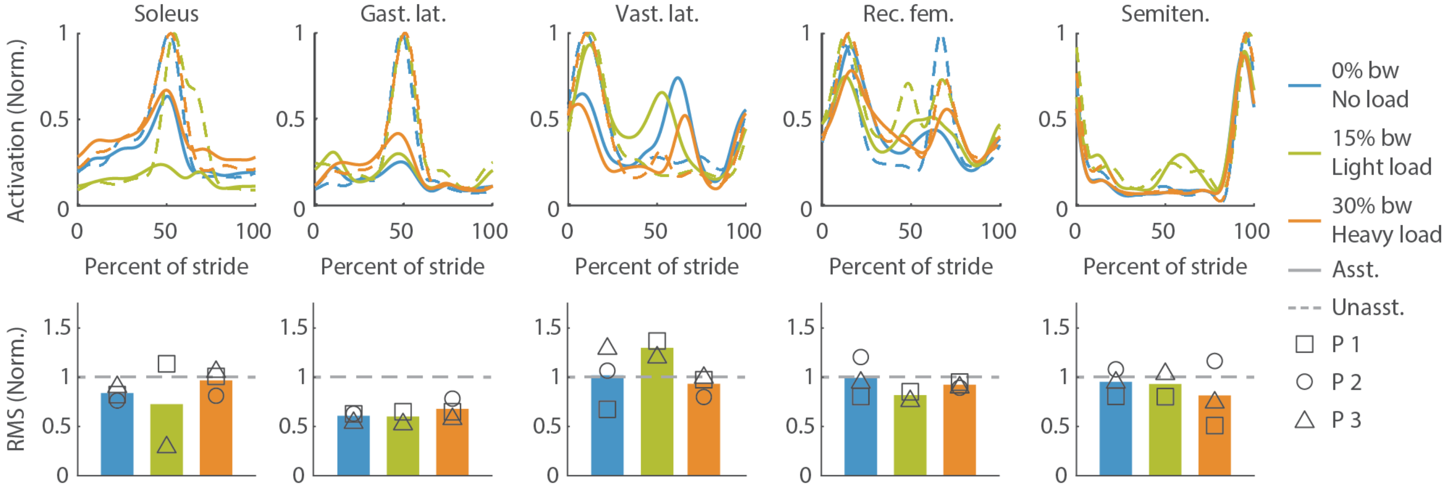

Figure 5. Average muscle activity profile over a stride (top row) and RMS of muscle activity (bottom row) without the minimum value subtracted. High frequency noise in the electromyography signal produce a vertical offset shown in the muscle activity profiles (top row). The offset can increase and decrease the RMS values (bottom row).

4. Kinematic results

Ground reaction forces

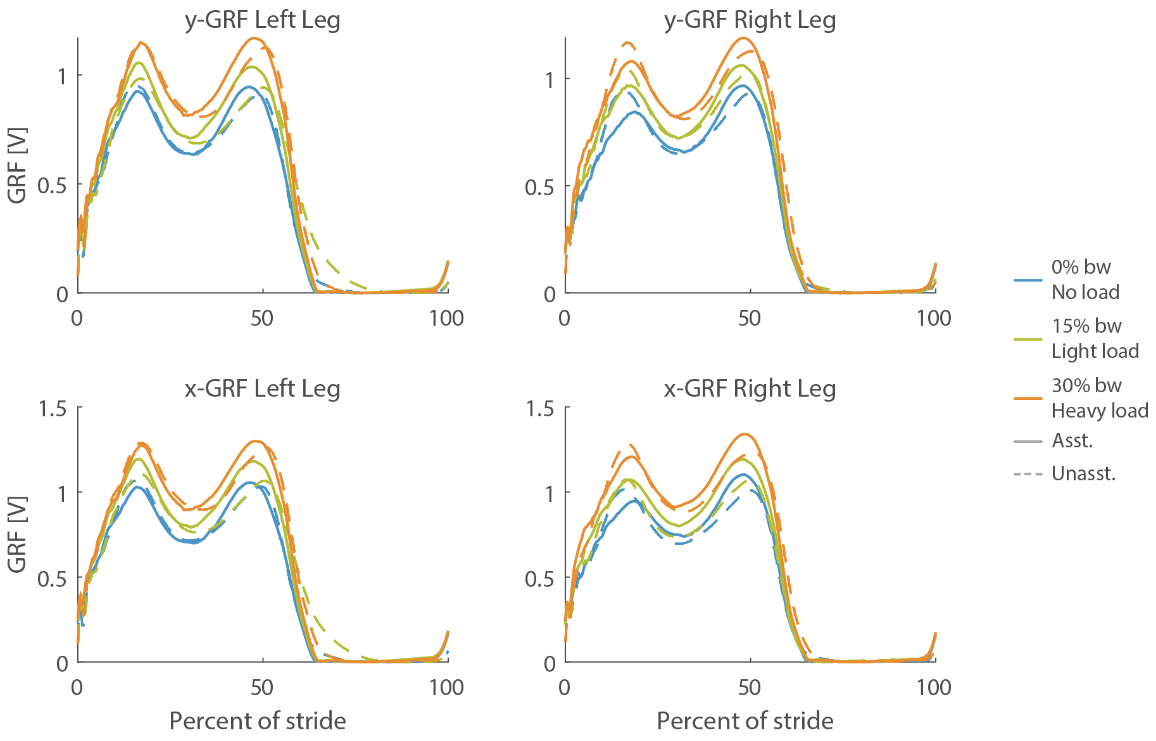

Figure 6. Ground reaction forces in the x and y directions for the left and right belts. No load (blue), light load (green) and heavy load (orange) results are shown. Both x and y ground reaction forces increased with load. Exoskeleton assistance typically decreased the magnitude of the first peak and increased the magnitude of the second.

Stride frequency

Table 8. Average stride frequency with no exoskeleton, unassisted and assisted. Stride frequency did not significantly change with load or exoskeleton condition.

Stride Frequency (Hz)	No load	Light load	Heavy load
No exo.	0.89 ± 0.08	0.89 ± 0.08	0.91 ±0.08
Unassisted	0.88 ± 0.08	0.89 ± 0.08	0.90 ± 0.09
Assisted	0.90 ± 0.06	0.92 ± 0.06	0.94 ± 0.09

5. Torque parameterization

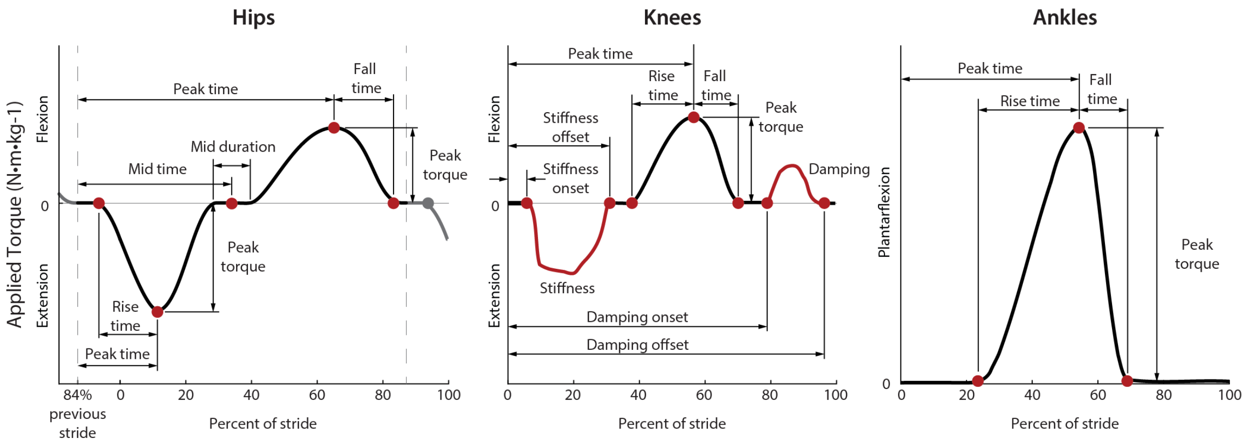

Figure 7. Parameterization of the hip, knee and ankle profiles.

The hip profile was defined by 8 parameters (Fig. 2 Hips). These parameters defined the rise time, peak time and peak magnitude of hip extension and the peak time, peak magnitude and fall time of hip flexion. A period of no torque was prescribed between extension and flexion periods and was defined by the mid-point timing and duration.

The knee profile was defined by 10 parameters (Fig. 2 Knees). It consisted of a virtual spring during stance, time based flexion torque near toe off, and a virtual damper during swing. The virtual spring was defined by the stiffness, onset time and offset time. The spring torque was the stiffness multiplied by the knee joint angle, which was set to zero when the knee was straight. Knee flexion torque near toe off was defined by the peak time, peak magnitude, rise time and fall time. The virtual damper during swing was parameterized with a damping coefficient, onset time and offset time, similar to the parameterization of the virtual spring.

The ankle profile was defined by 4 parameters similar to knee flexion near toe-off (Fig. 2 Ankles). These defined the peak time, peak magnitude, rise time, and fall time. Ankle torque was set to zero at 65% of stride at the latest to avoid torque application during swing. This constraint could shorten the fall time, for example a peak time of 55% of stride would result in a maximum fall time of 10% of stride.

6. Parameter ranges and optimized values

Hip parameter ranges and optimized values

The hip profile was defined by 8 parameters. It applied hip extension torque through heel strike, so the stride timer began at 84% of stride to avoid discontinuities in the desired profile at heel strike. To convert the hip timing parameters to be based off of heel strike, subtract 16% from the current value.

Table 9. Hip parameter ranges for all speeds and initial values for the no-load condition. The light load condition was initialized with the optimized values from the no-load condition, and the heavy load condition was initialized with the optimized values from the light load condition.

Hips	Hip ext. rise time	Hip ext. peak time	Hip ext. peak torque (Nm/kg)	Mid time	Mid dur.	Hip flex. Peak time	Hip flex. peak torque (Nm/kg)	Hip flex. fall time
**Min**	0.125	0.225	0.000	0.450	0.000	0.750	0.000	0.125
**Initial P1**	0.196	0.255	0.192	0.478	0.025	0.824	0.192	0.228
**Initial P2**	0.176	0.258	0.420	0.472	0.020	0.814	0.271	0.244
**Initial P3**	0.188	0.265	0.271	0.471	0.013	0.822	0.162	0.214
**Max**	0.250	0.300	0.600	0.525	0.100	0.850	0.500	0.300

Table 10. Optimized hip parameters for the no-load, light load, and heavy load conditions.

No load	HE RT	HE P time	HE torque	Mid time	Mid dur	HF P time	HF torque	HF FT
**P1**	0.187	0.260	0.211	0.473	0.014	0.800	0.207	0.203
**P2**	0.196	0.255	0.425	0.478	0.025	0.824	0.283	0.228
**P3**	0.178	0.266	0.326	0.468	0.008	0.808	0.176	0.220
**Average**	**0.187**	**0.260**	**0.321**	**0.473**	**0.015**	**0.811**	**0.222**	**0.217**
**Light load**								
**P1**	0.182	0.254	0.300	0.467	0.003	0.804	0.143	0.233
**P2**	0.211	0.246	0.536	0.494	0.024	0.819	0.255	0.231
**P3**	0.178	0.287	0.403	0.486	0.000	0.834	0.159	0.258
**Average**	**0.190**	**0.262**	**0.413**	**0.482**	**0.009**	**0.819**	**0.186**	**0.241**
**Heavy load**								
**P1**	0.190	0.254	0.312	0.468	0.000	0.797	0.313	0.217
**P2**	0.207	0.269	0.361	0.491	0.046	0.850	0.204	0.214
**P3**	0.189	0.294	0.496	0.475	0.000	0.841	0.087	0.260
**Average**	**0.196**	**0.272**	**0.390**	**0.478**	**0.016**	**0.829**	**0.201**	**0.231**

Knee parameter ranges and optimized values

The knee profile was defined by 10 parameters. The stride time is 0% at heel strike and 100% at the following heel strike of that leg. The knee profile has two state based periods, a virtual spring during stance and a virtual damper during late swing. These two periods were defined by the onset and offset timing of the periods and by the stiffness or damping constant. During the period of virtual spring torque, if the knee joint angle went to 0 before the end of the period, the exoskeleton stopped applying torque for participant comfort.

Table 11. Knee parameter ranges for all loads and initial values for the no-load condition. The light load condition was initialized with the optimized values from the no-load condition, and the heavy load condition was initialized with the optimized values from the light load condition.

Knees	Stiffness onset	Stiffness *k*	Stiffness offset	Flex. rise time	Flex. peak time	Flex. peak torque	Flex. fall time	Damping onset	Damping coefficient *b*	Damping offset
**Min**	0.001	0	0.2	0.15	0.525	0	0.05	0.725	0	0.9
**Initial P1**	0.0265	0.0073	0.2832	0.1651	0.6105	0.1587	0.0928	0.8074	1.1872	0.9848
**Initial P2**	0.025	0.008	0.271	0.205	0.588	0.247	0.094	0.812	1.763	0.968
**Initial P3**	0.026	0.008	0.289	0.164	0.602	0.124	0.094	0.801	0.915	0.967
**Max**	0.05	0.025	0.3	0.3	0.625	0.35	0.125	0.85	2.75	0.999

Table 12. Optimized knee parameters for no-load, light load, and heavy load conditions.

No load	KE k on	KE k	KE k Off	KF RT	KF P time	KF T	KF FT	Damp on	Damp *b*	Damp Off
**P1**	0.028	0.008	0.290	0.155	0.609	0.122	0.104	0.794	0.973	0.964
**P2**	0.027	0.016	0.283	0.165	0.611	0.203	0.093	0.807	1.348	0.985
**P3**	0.024	0.005	0.300	0.170	0.600	0.085	0.099	0.782	1.307	0.975
**Average**	**0.026**	**0.010**	**0.291**	**0.163**	**0.606**	**0.136**	**0.099**	**0.795**	**1.209**	**0.975**
**Light load**										
**P1**	0.028	0.005	0.280	0.156	0.604	0.084	0.098	0.782	1.915	0.966
**P2**	0.043	0.016	0.262	0.178	0.607	0.173	0.084	0.808	1.791	0.958
**P3**	0.035	0.000	0.297	0.188	0.596	0.078	0.108	0.746	1.037	0.980
**Average**	**0.035**	**0.007**	**0.280**	**0.174**	**0.603**	**0.112**	**0.097**	**0.778**	**1.581**	**0.968**
**Heavy load**										
**P1**	0.023	0.011	0.260	0.150	0.588	0.026	0.108	0.783	2.192	0.981
**P2**	0.042	0.025	0.235	0.169	0.607	0.080	0.086	0.803	0.739	0.999
**P3**	0.019	0.000	0.285	0.199	0.596	0.058	0.099	0.749	1.465	0.979
**Average**	0.028	0.012	0.260	0.173	0.597	0.054	0.097	0.778	1.465	0.986

Ankle parameter ranges and optimized values

The ankle profile was defined by 4 parameters. The stride time is 0% at heel strike and 100% at the following heel strike of that leg.

Table 13. Ankle initial values for the no-load condition and parameter ranges. The light load condition was initialized with the optimized values from the no-load condition, and the heavy load condition was initialized with the optimized values from the light load condition.

Ankles	Peak torque	Peak time	Rise time	Fall time*
**Min**	0.000	0.500	0.175	0.100
**Initial P1**	0.800	0.550	0.306	0.184
**Initial P2**	0.800	0.550	0.400	0.200
**Initial P3**	0.600	0.546	0.291	0.182
**Max (no load)**	0.800	0.550	0.400	0.200
**Max (loaded)**	0.900	0.550	0.400	0.200

*Torque was limited to be applied no later than 65% of stride, so, for example, if peak time was at its latest allowed value (55% of stride), fall time was limited to be 10% of stride.

Table 14. Optimized ankle parameters for no-load, light load, and heavy load conditions.

No load	Peak torque	Peak time	Rise time	Fall time*
**P1**	0.800	0.550	0.282	0.173
**P2**	0.800	0.550	0.306	0.190
**P3**	0.707	0.550	0.282	0.193
**Average**	0.769	0.550	0.290	0.186
**Light load**				
**P1**	0.900	0.550	0.252	0.173
**P2**	0.900	0.550	0.335	0.192
**P3**	0.831	0.548	0.287	0.182
**Average**	**0.877**	**0.549**	**0.291**	**0.182**
**Heavy load**				
**P1**	0.900	0.550	0.203	0.171
**P2**	0.900	0.550	0.382	0.184
**P3**	0.900	0.550	0.291	0.186
**Average**	0.900	0.550	0.292	0.180

7. Torque tracking

Table 15. Root mean square torque tracking error for the no-load, light load and heavy load conditions.The error is reported in Nm and as percent of the maximum torque. Knee assistance typically resulted in more torque tracking error. The state based periods have step changes in desired torque and change on a step to step basis. Iterative learning, part of the control structure, learns the torque tracking error over time, so it is slightly less effective when the desired torque changes on each step than it is with consistent desired torque.

**No load**	Hip	Knee	Ankle
**P1**	0.79 Nm (6.24%)	1.88 Nm (24.52%)	1.61 Nm (3.35%)
**P2**	1.13 Nm (2.96%)	3.48 Nm (19.31%)	0.90 Nm (1.25%)
**P3**	1.17 Nm (4.59%)	1.95 Nm (22.42%)	1.72 Nm (3.05%)
**Average**	1.03 Nm (4.60%)	2.43 Nm (22.08%)	1.41 Nm (2.55%)
**Light load**			
**P1**	1.11 Nm (6.17%)	2.54 Nm (26.89%)	1.65 Nm (3.05%)
**P2**	1.01 Nm (2.61%)	3.94 Nm (19.00%)	1.18 Nm (1.61%)
**P3**	1.02 Nm (3.17%)	1.41 Nm (16.36%)	1.58 Nm (2.37%)
**Average**	1.05 Nm (3.98%)	2.63 Nm (20.75%)	1.47 Nm (2.34%)
**Heavy load**			
**P1**	1.08 Nm (7.01%)	2.30 Nm (34.50%)	1.82 Nm (3.80%)
**P2**	1.11 Nm (3.72%)	3.78 Nm (17.51%)	1.06 Nm (1.62%)
**P3**	0.88 Nm (3.99%)	1.59 Nm (25.49%)	1.46 Nm (2.43%)
**Average**	1.02 Nm (4.91%)	2.56 Nm (25.83%)	1.45 Nm (2.62%)

8. Impact of the cloth mask

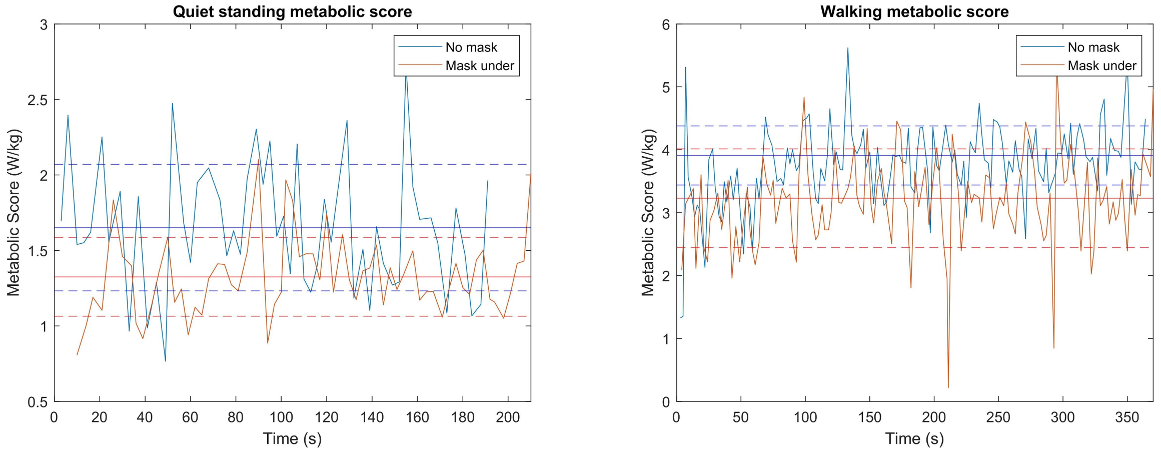

Figure 8. Metabolic impact of the cloth mask under the metabolic mask. Participant 1 measured their metabolic cost of quiet standing and walking on the treadmill for 6 minutes each. All measurements were collected in the same session. They measured their metabolic cost with no cloth mask (blue) and with a cloth mask under the metabolics mask (red). The average of the last 3 minutes of walking is shown with the solid line, and the dashed lines show the range for one standard deviation. The participant was the only person in the lab space, so they did not put others at risk by not wearing a mask. The cloth mask lowered the metabolic cost by 0.33 W/kg for the standing condition and by 0.68 W/kg for the walking condition.

Table 16. Metabolic cost (W/kg) for Participant 1 with and without the cloth mask during the no load condition

P 1	Quiet standing	No exoskeleton	No torque	Optimized torque	Percent reduction
No Cloth Mask	1.77 ± 0.01	5.18 ± 0.06	6.07 ± 0.12	3.78 ± 0.06	53%
Cloth Mask	1.63 ± 0.08	4.77 ± 0.08	6.04 ± 0.41	3.48 ± 0.10	58%
Difference	0.14	0.41	0.03	0.30	5%

Table X. Metabolic cost (W/kg) for Participant 2 with and without the cloth mask during the no load condition

P 2	Quiet standing	No exoskeleton	No torque	Optimized torque	Percent reduction
No Cloth Mask	1.33 ± 0.02	3.88 ± 0.09	4.56 ± 0.14	2.92 ± 0.11	51%
Cloth Mask	0.86 ± 0.06	2.97 ± 0.14	3.30 ± 0.01	2.09 ± 0.20	51%
Difference	0.47	0.91	1.26	0.83	0%
